# Antibiotic consumption trends in Ghana: analysis of six-years pharmacy issue data from a secondary healthcare facility

**DOI:** 10.1093/jacamr/dlad025

**Published:** 2023-03-21

**Authors:** Appiah-Korang Labi, Bridget S Kartey, George Kwesi Hedidor, Benjamin Demah Nuertey, Elsie Kodjoe, Leslie No Vanderpuije, Noah Obeng-Nkrumah

**Affiliations:** Department of Medical Microbiology, University of Ghana Medical School, University of Ghana, Korle-Bu, Accra, Ghana; Life Course Cluster, WHO Ghana Country Office, No. 7 Ameda Street, Roman Ridge, Accra, Ghana; Pharmacy Department, Eastern Regional Hospital, Koforidua, Ghana; Life Course Cluster, WHO Ghana Country Office, No. 7 Ameda Street, Roman Ridge, Accra, Ghana; Life Course Cluster, WHO Ghana Country Office, No. 7 Ameda Street, Roman Ridge, Accra, Ghana; Life Course Cluster, WHO Ghana Country Office, No. 7 Ameda Street, Roman Ridge, Accra, Ghana; Pharmacy Unit, Ghana Health Service Eastern Regional Health Directorate, Koforidua, Ghana; Department of Medical Laboratory Science, School of Biomedical and Allied Health Sciences, University of Ghana, Accra, Ghana

## Abstract

**Background:**

Surveillance of hospital antibiotic consumption provides data to inform corrective action and for monitoring antimicrobial stewardship activities. This study described antibiotic consumption patterns from 2016 through 2021 at a secondary healthcare facility in Ghana.

**Methods:**

Using the WHO methodology for surveillance of antimicrobial consumption in hospitals, we analysed a 6-year pharmacy issue data at the Eastern Regional Hospital. We report on the defined daily dose (DDD) per 100 patients, types of antibiotics consumed according to Anatomic Therapeutic Classification (ATC), WHO AWaRe classification; trends in antibiotic consumption and expenditure per DDD of antibiotics consumed.

**Results:**

Over the period, the mean (±standard deviation) antibiotic consumption rate was 256.7 ± 33 DDD/100 patients per year. A linear regression model showed an insignificant decreasing trend in antibiotic consumption (coefficient for time –0.561; *P* = 0.247). The top three consumed antibiotics in DDD/100 patients at ATC level 5 were amoxicillin-clavulanate (*n* = 372.6), cefuroxime (*n* = 287.4) and sulfamethoxazole-trimethoprim (*n* = 145.8). The yearly Access-to-Watch ratio decreased from 2.4 in 2016 to 1.2 in 2021. The mean yearly cost of antibiotics was $394 206 ± 57 618 US dollars. The top three antibiotics consumed in terms of cost were clindamycin $718 366.3, amoxicillin-clavulanate $650 928.3 and ceftriaxone $283 648.5.

**Conclusion:**

This study showed a sturdy rate of antibiotic consumption over the 6-year period with a year-on-year decrease in the Access-to-Watch antibiotic ratio. Data from pharmacy drug issues offer an opportunity to conduct antibiotic consumption surveillance at the hospital and national level in Ghana.

## Introduction

Globally, there has been a 65% increase in antimicrobial consumption (AMC) in humans between the years 2000 and 2015, with this increase driven mainly by low- and middle-income countries.^1^ Increasing antimicrobial use is considered a major driver of antimicrobial resistance (AMR). AMR is considered a global public health problem.^2,3^

Several reports from Ghana show increasing AMR, with a high prevalence of resistance phenotypes such as extended-spectrum beta-lactamases and increasing reports of carbapenemase-producing bacteria.^4–7^ The Ghana national policy and action plan on AMR that is aligned with the global action plan on AMR highlight the need to strengthen surveillance and research as well as optimize the use of antimicrobials.^8^ Optimizing antimicrobial use and consumption through antimicrobial stewardship hinges on the availability of antimicrobial use and consumption data. Antimicrobial use and consumption data can provide warning signs concerning antimicrobial exposure and use, allowing for the institution of corrective measures. It may also serve as a monitoring tool for interventions aimed at reducing antimicrobial exposure.^1,9^ In Ghana, surveillance of antimicrobial consumption at the hospital and national level is lacking, however, several point prevalence surveys on antimicrobial use have shown 47% to 66% prevalence of antimicrobial use among hospitalized patients.^10–13^ Currently, Ghana is undergoing a national digitalization agenda that includes the provision and use of electronic health records in healthcare facilities. Electronic health records of hospital pharmacies and doctors’ prescribing notes represent low-hanging fruit for conducting surveillance of antimicrobial use and consumption at the hospital level in Ghana.

The Eastern Regional Hospital (ERH),^14^ is a secondary referral facility that provides a range of specialist services for the >2 million people living in rural and urban settings in the Eastern region of Ghana.^15^ The hospital has a history of electronic health records use for the past 6 years and has accumulated records useful for conducting antimicrobial consumption studies. These data could provide an understanding of one of the potential drivers of AMR in the hospital and inform antimicrobial stewardship strategies. In this study, we evaluated the antibiotic consumption trends at the ERH from 2016 through 2021 using WHO’s defined daily dose (DDD) methodology.

## Materials and methods

### Study design and settings

Using the WHO methodology for the global programme on surveillance of antimicrobial consumption in hospitals,^16,17^ we analysed a 6-year pharmacy central pharmacy issues data. The central pharmacy stores of the hospital dispense drugs to pharmacy units associated with various departments of the hospital. The Pharmacy Department distributes drugs using the stock system, where dispensed drugs through the supply chain are recorded. Patients pay for any drugs consumed with cash or through the National Health Insurance System. A recent point prevalence survey conducted at the hospital showed an antimicrobial use prevalence of 50%.^13^ The hospital uses the national standard treatment guidelines as the main document supporting antimicrobial prescriptions although other documents such as the British National Formulary are used.^18^ The ERH has a microbiology laboratory that routinely performs bacterial cultures and antimicrobial susceptibility testing. The hospital’s Information Technology Department manages electronic health records where medical records of patients and pharmacy data are stored.

### Antibiotic inclusion criteria

Data summaries on antibiotics dispensed over 71 consecutive months from January 2016 through November 2021 were copied from pharmacy electronic records and transferred to an Excel^™^-based data collection instrument. Data extracted included the antibiotic name, the dosage, route of administration, quantity, date dispensed and the cost of the issued antibiotics. No patient-level information on prescriptions—e.g. indication or age of the patient—was available. Total patient attendance to the hospital for the years under consideration was retrieved. In this study, we included only data on antibiotics for systemic use, classified in the J01 category, by the WHO Anatomic Therapeutic (ATC) classification system.^17^ The WHO also recommends categorizing antibiotics as Access, Watch or Reserve (AWaRe),^19,20^ and we used the 2021 database^20^ to classify them accordingly. Topical antibiotics were excluded from the study. Antimicrobials used for managing tuberculosis, parasites and fungi were not also included.

### Calculation of antibiotic consumption rates

Antibiotic consumption was estimated on the basis of the amount dispensed from the central pharmacy store in standard units of milligrams or millilitres. Each standard unit was defined based on a single tablet, capsule, ampoule, vial or liquid preparation for oral consumption.^16^ Each antibiotic was assigned to a WHO ATC level 4 and 5 classifications. We then expressed the number of drugs dispensed for each antibiotic class as our consumption data in DDD according to WHO methodology.^16^ The DDD is the assumed average maintenance dose per day for a medicine used for its main indication in an adult. It is a globally accepted unit for measuring drug consumption of different strengths or combinations and can be used to compare rates between different antibiotic categories and years.^19,20^ The DDD was calculated by converting the total amount of antibiotic dispensed in into grams and divided by the standard WHO ATC DDD value given in grams. When measuring antibiotic consumption in a hospital, where attendance data is available but no patient-level information can be accessed, DDD per 100 patients is the recommended method for standardization.^16^ In this study, we used total patients’ attendance at ERH to represent the population served by the hospital. This was the closest and best representation of the population served by the hospital, as most in- and outpatients are accounted for in the patient attendance data. The DDD/100 patients were obtained by dividing the calculated total DDD for each year by the patient attendance for the respective year and multiplying the ratio by 100. The expenditure on antibiotic consumption was calculated as expenditure per DDD by multiplying the number of antibiotics issued in a period (e.g. all antibiotics or specific types) and the cost and dividing by the calculated DDD of the antibiotic for that period. For subgroup analysis, we compared yearly variations in antibiotic consumption for drug categories (e.g. AWaRe) by calculating the per cent contribution of that antibiotic category to the overall antibiotic consumption that year.

### Data analysis

Data were entered and cleaned using Microsoft Excel^®^ 2021 and exported to the STATA^®^ software for descriptive and analytical studies. Continuous variables were presented as means ± standard deviations (SD) and sums. Categorical variables were presented as frequencies and percentages. Difference in antibiotic consumption across the years was determined using Chi-square test, with the Marascuilo’s *post hoc* procedure applied for subsequent pairwise comparisons. The Chi-square trend analysis was used to compare the trend in consumption of WHO AWaRe antibiotic groups over the years. Measures of relative consumption, expressed as a percentage of the total consumption of groups of antibiotics, were derived for each antibiotic. A linear regression model was used to assess the trend of antibiotic consumption rate over time. The coefficient for time and *P* value for the trend of antibiotic consumption was calculated using yearly measures. All statistical tests were considered significant at a *P* value <0.05. The cumulative change in consumption for each antibiotic over the study period was calculated by adding the absolute differences in DDD/100 patients between 2017 and 2016, 2018 and 2017, 2019 and 2018, 2020 and 2019, and 2021 and 2020. With regards to the cost of consumed antimicrobial agent in US dollars ($), the amount of antimicrobial agent in Ghana cedi was converted to dollars using the average yearly dollar rate.

### Ethical considerations

Ethical clearance for the study was obtained from the Ghana Health Service Ethics Review Committee with protocol number GHS-ERC 004/05/22. All data extracted from the EHR were summaries and aggregated data from antimicrobials issued from the pharmacy stores between 2016 and 2021. No individual patient records were collected, thus findings from the study cannot be linked to any patient.

## Results

Over the 6-year survey period, we extracted data summaries on 15 different antibiotics at ATC level 4 (yearly mean ± SD, 14.33 ± 0.52) and 26 antibiotics at ATC level 5 (yearly mean ± SD, 24.67 ± 0.82) from the electronic records at ERH for analysis (Table 1). From a total of 26 types of antibiotics issued, 13 were in the Access category, 10 in the Watch category and none were in the Reserve category. The antibiotics were issued to a yearly mean population of 170 044.2 ± 10 878.5 patients attending the hospital. The mean volume in DDD of antibiotics issued per year was 81 839.2 ± 190 733.4. Adult antibiotics, compared to paediatric formulations, accounted for 94.5% (*n* = 1455.46/84.5) of the total volume in DDD of antibiotics issued over the 6 years.

**Table 1.** Summary of hospital metrics and daily defined doses

Summaries	2016	2017	2018	2019	2020	2021	Grand total
	Hospital metrics						
Total patient visits (%)	155 560 (15.1)	161 084 (15.7)	177 969 (17.3)	192 428 (18.7)	163 336 (15.9)	177 639 (17.3)	1 028 016 (100)
Hospital admissions (%)	16 350 (16.7)	16 348 (16.7)	16 340 (16.7)	16 326 (16.7)	16 324 (16.7)	16 332 (16.7)	98 020 (100)
Number of ATC level 4 antibiotics prescribed	14	14	14	14	15	15	15
Number of ATC level 5 antibiotics prescribed	24	25	24	26	24	25	26
Distinct Access antibiotics prescribed	11	11	13	12	10	11	13
Distinct Watch antibiotics prescribed	8	9	10	8	8	9	10
Distinct Reserve antibiotics prescribed	0	0	0	0	0	0	0
Distinct unclassified antimicrobials prescribed	0	0	0	1	0	1	1
	Volume in DDD (%) of antibiotics issued per year						
All antibiotics	397 276.6	497 341.3	514 471.3	422 362.0	361 231.0	437 103.4	2 629 786.0
Access antimicrobial	280 071.0 (70.5)	337 892.9 (67.9)	325 579.4 (62.4)	251 884.9 (59.6)	222 921.8 (61.7)	239 780.1 (54.9)	1 658 130.0 (63.3)
Watch antimicrobials	117 205.6 (29.5)	159 448.4 (32.1)	188 891.9 (36.7)	170 469.6 (40.4)	138 309.2 (38.3)	197 283.2 (45.1)	971 608.0 (36.7)
Reserve antimicrobials	0	0	0	0	0	0	0
Unclassified antimicrobials	0	0	0	7.5 (0.002)	0	40.0 (0.01)	47.5 (0.002)
Oral antimicrobials	350 764.1 (88.3)	454 502.5 (91.4)	464 406.0 (90.3)	360 646.8 (85.4)	298 642.7 (82.7)	375 286.9 (85.9)	2 304 249.0 (87.7)
Parenteral antimicrobials	46 512.5 (11.7)	42 838.8 (8.6)	50 065.3 (9.7)	61 715.2 (14.6)	62 588.3 (17.3)	61 816.4 (14.1)	325 536.4 (12.3)
Generic antimicrobials	396 545.1 (99.8)	496 467.0 (99.8)	508 526.8 (98.8)	413 674.9 (97.9)	355 647.8 (98.5)	427 825.3 (97.9)	2 598 687 (98.9)
Originator antimicrobials	731.5 (0.2)	874.3 (0.2)	5944.5 (1.2)	8687.1 (2.1)	5583.2 (1.5)	9278.0 (2.1)	31 098.6 (1.1)
Paediatric formulation	25 797.5	26 633.8	28 363.3	24 635.2	13 427.3	25 552.2	144 409.4 (5.5)
Adult formulation	371 479.1	470 707.5	486 108.0	397 726.7	347 803.7	411 551.2	2 485 376.0 (94.5)
Access-to-Watch ratio	2.4	2.1	1.7	1.5	1.6	1.2	

*%. Percentages: unclassified antimicrobials include Ceftriaxone sulbactam and Ciprofloxacin tinidazole.

### Total antibiotic consumption rate

**Figure 1.**
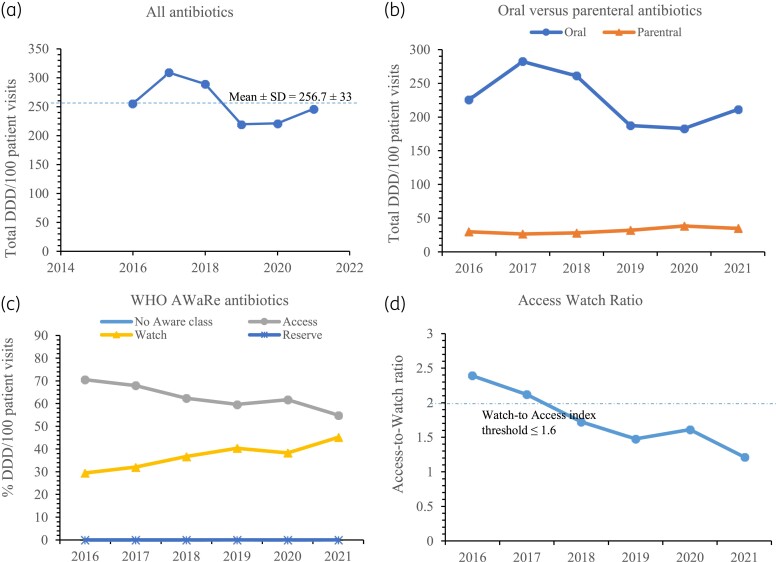
Trends in antibiotic consumption rates in DDD/100 patient visits for: (a) all antibiotics, (b) oral versus parenteral antibiotics, (c) WHO AWaRe antibiotics and (d) Access-to-Watch antibiotic index with trend line that shows the WHO recommended ratio threshold of ≤1.6.

Figure 1(a) shows the antibiotic consumption in DDD/100 patients for each year. The mean antimicrobial consumption rate for all antibiotics over the 6 years was 256.7 ± 33 DDD/100 patients. The highest consumption of 308.7 DDD/100 patients was recorded in 2017 and the lowest of 219.5 DDD/100 patients was recorded in 2019. A linear regression model used to assess the pattern of antibiotic consumption over time showed an insignificant decreasing trend (coefficient for time −0.561; *P* = 0.247). Differences in annual antibiotic consumption levels were not significant for the 6 years under study (*χ*^2 ^= 6.409, *P* = 0.1706). When follow-up pairwise comparisons were conducted with Marascuilo’s *post hoc* procedure, none of the annual variations in antibiotic consumption between any pair of years from 2016 to 2021 was significant (*P* > 0.05 for all 15 pairwise comparisons). Of the total antibiotics consumed DDD/100 patients, 87.7% (*n* = 1354/1543.9) were oral agents. Figure 1(b) shows a clear separation in which the consumption trend in DDD/100 for oral antibiotics from 2016 through 2021 mirrors the consumption levels for all antibiotics [Figure 1(a)] and exceeds the consumption rate for parenteral antibiotics by several folds by a factor of >5 across the study years. Overall, ∼94.5% (*n* = 1526.4/1543.9) of the total consumed antibiotics were generic brands.

### Antimicrobial consumption according to AWaRe classifications

Most (63.3%, *n* = 564.79/975.11) of the antibiotics consumption in DDD/100 patients over the 6 years belonged to the Access group and there was no consumption of Reserve antibiotics over the period of study. Unclassified antibiotics represented 0.026 DDD/100 patients accounting for 0.002% of total consumed antibiotics. Figure 1(c) shows the trend in yearly antibiotic consumption in DDD/100 patients for the WHO AWaRe classification groups. There was a significant yearly increase (*χ*^2^ trend, *P* = 0.0001) in the consumption rate of Watch antibiotics from 2016 to 2021 and a commensurate progressive decrease in consumption of Access class of antibiotics (*χ*^2^ trend, *P* = 0.0001). For example, in 2016, 70.5% (*n* = 180.1/255.4) of the total DDD/100 patients belonged to Access group of antibiotics and this decreased to 54.9% (*n* = 135.0/246.1) in 2021. In contrast, the proportion of Watch antibiotics contributing to total DDD/100 patients was 29.5% (*n* = 73.4/255.4) in 2016 and increased significantly over the years to 45.1% (*n* = 111.1/246.1) in 2021. The Access-to-Watch ratio, based on antibiotic consumption rate in DDD/100 patients, versus the year graph is displayed in Figure 1(d). There was a decrease in the Access-to-Watch ratio across the years from 2.4 in 2016 to 1.2 in 2021. Among the Watch group of antibiotics, the top five with the highest consumption rate in DDD/100 patients over the 6 years were cefuroxime (*n* = 287.4), azithromycin (*n* = 88.4), ciprofloxacin (*n* = 82.9), ceftriaxone (*n* = 41.9) and clarithromycin (*n* = 39.9) (Table S1, available as Supplementary data at *JAC* Online). The top five Access antibiotics with the highest DDD/100 patients were amoxicillin-clavulanate (*n* = 372.6), sulfamethoxazole-trimethoprim (*n* = 145.8), metronidazole (*n* = 127.8), doxycycline (*n* = 95.8) and clindamycin (*n* = 91.9).

### Antimicrobial consumption rates by ATC classification

**Figure 2.**
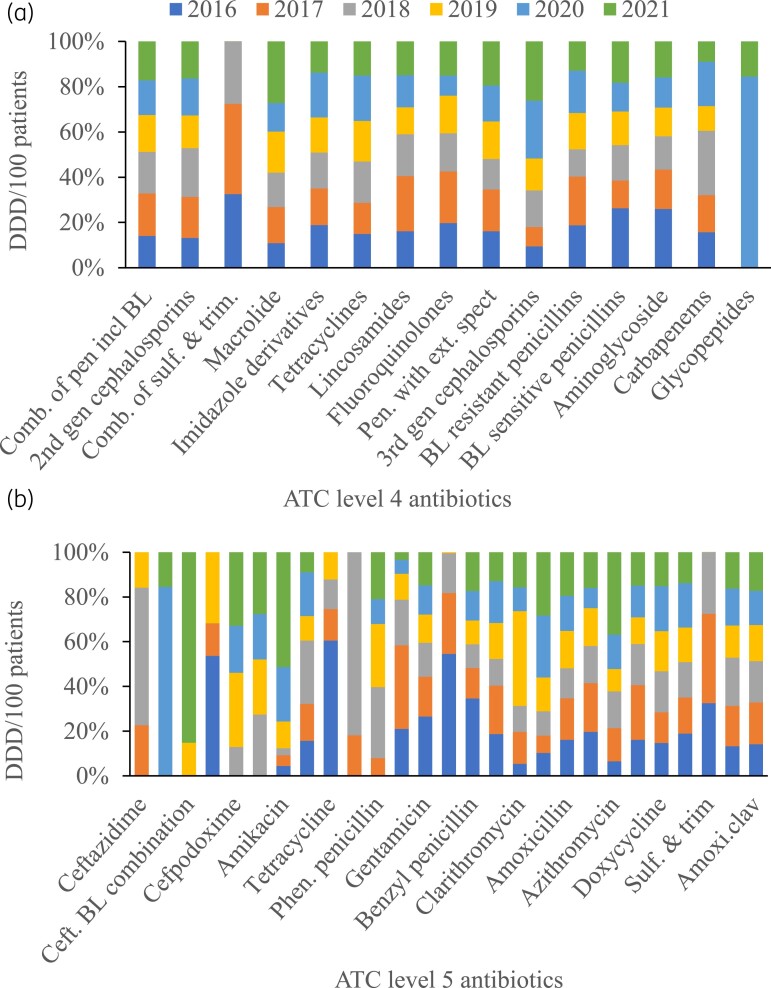
Antibiotic consumption at DDD/100 patients for Anatomic Therapeutic Classification (ATC): (a) Level 4 and (b) Level 5. *Comb. of pen incl BL, combination of penicillin including beta-lactams; Comb. of sulf. & trim., combination of sulphonanmides and trimethoprim derivatives; Sulf. & trim., sulphurmethoxazole and trimethoprim; Pen. with ext. spect, penicillin with extended-spectrum; BL, beta-lactamase; Ceft. BL combination, ceftriaxone beta-lacatamse combination; Phen. penicillin, Phenoxylmethyl penicillin; Amoxi.clav., amoxicillin-clavulanic acid.

The DDD/100 patients according to ATC level 4 is presented in Figure 2(a). The top five consumed antibiotics are over the six-year period were penicillins with beta-lactamase combinations (*n* = 372.6.2), second-generation cephalosporins (*n* = 287.4), combinations of sulfonamides and trimethoprim including derivatives (*n* = 145.8), macrolides (*n* = 142.3) and imidazole derivatives (*n* = 127.9). The complete list of antibiotics at ATC level 4 and their yearly consumption rates at can be found in Table S2. At ATC level 5, the top five antibiotics with the highest consumption rate in DD/100 patients were amoxicillin-clavulanate (*n* = 372.6), cefuroxime (*n* = 287.4), sulfamethoxazole-trimethoprim (*n* = 145.8), metronidazole (*n* = 127.9), and doxycycline (*n* = 95.9) [Figure 2(b)]. Suffice to say that while amoxicillin-clavulanate remained the most consumed antimicrobial yearly, there were yearly variations in the positions of the other top five antibiotics as shown in Table S3.

### Cumulative changes in antibiotic consumption

**Figure 3.**
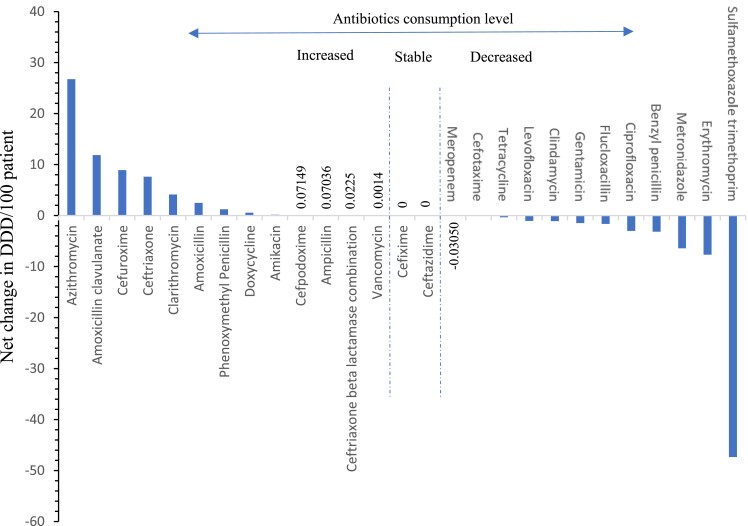
Cumulative increase and decrease in antibiotic ATC level 5 consumption from 2016 to 2021.

Figure 3 shows the 6-year cumulative change in ATP level 5 antibiotic consumption. Cefixime and ceftazidime were the only antibiotics with zero change in consumption DDD per 100 patients from 2016 through 20121. Other antibiotics including meropenem (−0.03 DDD/100 patients), vancomycin (0.001 DDD/100 patients) and amikacin (0.16 DD/100 patients) displayed only marginal changes in DDD/100 patients. Azithromycin showed the highest net increase in consumption (26.78 DDD/100 patients), followed by amoxicillin-clavulanate (11.84 DDD/100 patients), cefuroxime (8.92 DDD/100 patients) and ceftriaxone (7.60 DDD/100 patients). In contrast, other antibiotics recorded a net decrease in consumption. The top four antibiotics with the highest drop in consumption were sulfamethoxazole-trimethoprim (−47.33 DDD/100 patients), erythromycin (−7.65 DDD/100 patients), metronidazole (−6.42 DDD/100 patients) and benzyl-penicillin (−3.17 DDD/100 patients).

### Cost of antimicrobials consumed

The total cost of antibiotics over the 6 years was $2 365 241 with a yearly mean cost of $394 206 ± 57 618. The least gross amount of $301 011.7 was spent on antibiotics in 2020 and the highest amount of $494 934 was spent in 2018. The yearly average cost of antibiotics per DDD was $0.89 (SD 0.06), ranging from $0.83 in 2020 to $0.95 in 2016. Patients attending the ERH spent a mean of $2.3 ± 0.3 on antibiotics per visit to the hospital. The top five antibiotics contributing to overall antibiotic cost over the 6-year period were clindamycin ($718 366.3), amoxicillin-clavulanate ($650 928.3), ceftriaxone ($283 648.5), cefuroxime ($262 708.9) and metronidazole ($80 258.5).

## Discussion

Globally, antibiotic consumption has increased over the last two decades, fuelled mainly by consumption in LMICs.^1^ Data on antibiotic consumption are critical in informing and evaluating antimicrobial stewardship programmes implemented at the hospital and national levels to combat AMR. This study found an insignificant drop in antibiotic consumption from 2019 to 2021 compared to 2016 to 2018, with the most consumed antimicrobial in the hospital being amoxicillin-clavulanate.

Antibiotic consumption in the hospital increased from 2016 to 2018 and decreased sharply between 2019 and 2020 before beginning to rise again in 2021. Whereas the drop in 2020 antibiotic consumption may be attributed to the COVID-19 pandemic and the consequent drop in hospital attendance, it is not easy to fathom the sharp drop in antibiotic consumption in 2019. One possible reason could be stockouts; however, this study did not have access to this data. Amoxicillin-clavulanate and cefuroxime were the two commonest antibiotics consumed in the hospital over the study period. Similarly, these agents have been documented as part of the top five antibiotics used in hospitalized patients from previous point prevalence surveys in Ghana.^10,13^ These antibiotics are recommended for the treatment of upper and lower respiratory tract and urinary tract infections by the Standard Treatment Guidelines of Ghana,^18^ and they are on the national health insurance of Ghana’s list of drugs,^21^ so are not prohibited by out-of-pocket cost. On the contrary, agents like ceftriaxone which feature prominently as part of the top five antibiotics used in most point prevalence surveys in Ghana was the tenth most consumed antibiotic in this study.^10,13^ Also, there was sharp rise in the consumption of azithromycin in 2021. This could be due to increased use as a result of speculations that suggested azithromycin as an agent for treating COVID-19. This highlights the important role of antibiotic consumption surveillance or studies in its ability to give a complete antibiotic utilization picture compared to point prevalence surveys. It is of interest to note, however, that other antibiotics such as clindamycin and ceftriaxone were responsible for the first and third antibiotics when expenditure was assessed.

Overall, most antibiotics prescribed belonged to the Access group of antibiotics, however, the proportion of Watch antibiotics consumed yearly increased from 2016 to 2021 and is exemplified by a decreasing Access-to-Watch index from 2.4 in 2016 to 1.2 in 2021. This means that as of 2021 antibiotic consumption in the hospital did not conform to the WHO target, which requires at least 60% of overall antibiotic consumption to be made of the Access group of antibiotics.^20^ This rise in Watch antibiotic use relative to the Access group of antibiotics is similar to global findings especially those from lower- and middle-income countries (LMICs) and may be a reflection of increasing antibiotic resistance observed in the study setting as has been suggested elsewhere.^22^ Increasing the use of Watch antibiotics could also be attributed to improved economic status in many LMICs with an associated increased purchasing power for more expensive broad-spectrum antibiotics,^1^ and uncertainty concerning febrile illness diagnosis. ^23^ There was no consumption of reserve antibiotics in the hospital over the study period, this may be a result of their unavailability on the local market, absence in the Standard Treatment Guidelines of Ghana^18^ and the fact that they are not funded by the health insurance scheme ^21^. Similar absent use of reserve antibiotics has been documented in point prevalence surveys among hospitalized patients in Ghana.^10,13,24,25^

This study has potential limitations. First, the study is limited to one secondary healthcare facility thus finding may not be generalizable to other healthcare settings. Second, the data could not be segregated into outpatient and inpatient status that would have been useful to show any differences in antibiotic consumption patterns. However, our data showed that 90% of antibiotics were oral formulations, which suggests that a large proportion of consumed antibiotics was prescribed to outpatients. Antibiotic consumption data for 2021 was short by 1 month due to a change in EHR at the hospital, and this may have affected the overall antibiotic consumption observed especially for that year. The level of antibiotic resistance in the population under study is unknown and this could have given better meaning to the observed antibiotic consumption. Also, the findings in this study are not commensurate with the appropriateness of use. This study used hospital antibiotics issue data that are dependent on the medications stocked by the hospital and did not account for medications that were prescribed and purchased in pharmacies outside the hospital for use in the hospital by individual patients. This could explain the lack of data on reserve antibiotics that were not stocked by the hospital. Also, the data collected did not account for stockouts that could have affected consumption levels. However, findings from this study are useful because it is one of the first to describe hospital-wide antibiotic consumption in Ghana and describes the potential use of drug issuance data as a measure of hospital-level antibiotic consumption. These data could serve as baseline data for future studies and antibiotic stewardship initiatives.

This study has the following policy implications. First, data from pharmacy drug issues may be a useful resource for conducting antibiotic consumption surveillance at the hospital and national level and may represent low-hanging fruit for consumption surveillance in Ghana considering the EHR rollout across healthcare facilities. Second, there is a need to understand the reasons behind the decreasing Access-to-Watch ratio observed in this study through the conduct of further studies. This will be important in influencing deliberate policies aimed at reversing the trend towards >60% prescription of antibiotics in the hospital belonging to the Access group.^20^ Third, there is also the need to strengthen AMR surveillance activities at the hospital to monitor the impact of the increase in the use of Watch antibiotics.

## Conclusion

This study showed a sturdy rate of antibiotic consumption over the 6-year study period with a year-on-year decrease in the Access-to-Watch ratio of antibiotics. Amoxicillin-clavulanate, cefuroxime and sulfamethoxazole-trimethoprim were the three most consumed antibiotics; however, clindamycin, amoxicillin-clavulanate and ceftriaxone were the top three antibiotics according to the total budget spent. Data from pharmacy drug issues offer an opportunity to conduct antibiotic surveillance at the hospital level in Ghana and when aggregated may give a national picture. This could be a useful resource for monitoring antibiotic stewardship activities at the hospital and national levels.

## Supplementary Material

dlad025_Supplementary_Data

## Data Availability

The data for this study are available upon reasonable request from the corresponding author.

## Supplementary data

Tables S1 to S3 are available as Supplementary data at *JAC* Online.

## References

[1] Klein EY , Van Boeckel TP, Martinez EM et al Global increase and geographic convergence in antibiotic consumption between 2000 and 2015. Proc Natl Acad Sci U S A 2018; 115: E3463-70. 10.1073/pnas.1717295115 29581252 PMC5899442

[2] Holmes AH , Moore LS, Sundsfjord A et al Understanding the mechanisms and drivers of antimicrobial resistance. Lancet 2016; 387: 176–87. 10.1016/S0140-6736(15)00473-0 26603922

[3] Goossens H . Antibiotic consumption and link to resistance. Clin Microbiol Infect 2009; 15: 12–5. 10.1111/j.1469-0691.2009.02725.x 19366364

[4] Banu RA , Alvarez JM, Reid AJ et al Extended spectrum beta-lactamase *Escherichia coli* in river waters collected from two cities in Ghana, 2018–2020. Trop Med Infect Dis 2021; 6: 105. 10.3390/tropicalmed6020105 34203078 PMC8293421

[5] Codjoe FS , Donkor ES, Smith TJ et al Phenotypic and genotypic characterization of carbapenem-resistant gram-negative *Bacilli* pathogens from hospitals in Ghana. Microb Drug Resist Larchmt N 2019; 25: 1449–57. 10.1089/mdr.2018.0278 31237486

[6] Labi A-K , Nielsen KL, Marvig RL et al Oxacillinase-181 carbapenemase-producing *Klebsiella pneumoniae* in neonatal intensive care unit, Ghana, 2017–2019. Emerg Infect Dis 2020; 26: 2235–8. 10.3201/eid2609.200562 32818427 PMC7454046

[7] Obeng-Nkrumah N , Twum-Danso K, Krogfelt KA et al High levels of extended-spectrum beta-lactamases in a major teaching hospital in Ghana: the need for regular monitoring and evaluation of antibiotic resistance. Am J Trop Med Hyg 2013; 89: 960–4. 10.4269/ajtmh.12-0642 24043693 PMC3820343

[8] Ministry of Health . Ghana National Action Plan on antimicrobial resistance (2017–2021). 2017. https://www.moh.gov.gh/wp-content/uploads/2018/04/NAP_FINAL_PDF_A4_19.03.2018-SIGNED-1.pdf

[9] Saleem Z , Hassali MA, Godman B et al Point prevalence surveys of antimicrobial use: a systematic review and the implications. Expert Rev Anti Infect Ther 2020; 18: 897–910. 10.1080/14787210.2020.1767593 32394754

[10] Labi A-K , Obeng-Nkrumah N, Nartey ET et al Antibiotic use in a tertiary healthcare facility in Ghana: a point prevalence survey. Antimicrob Resist Infect Control 2018; 7: 15. 10.1186/s13756-018-0299-z 29423190 PMC5787245

[11] Labi A-K , Obeng-Nkrumah N, Sunkwa-Mills G et al Antibiotic prescribing in paediatric inpatients in Ghana: a multi-centre point prevalence survey. BMC Pediatr 2018; 18: 391. 10.1186/s12887-018-1367-5 30572851 PMC6302438

[12] Labi A-K , Obeng-Nkrumah N, Owusu E, et al Multi-centre point prevalence survey of hospital-acquired infections in Ghana. J Hosp Infect 2019; 101: 60–68. https://www.sciencedirect.com/science/article/pii/S0195670118302573. 10.1016/j.jhin.2018.04.019 29730140

[13] Labi A-K , Obeng-Nkrumah N, Dayie NTKD et al Antimicrobial use in hospitalized patients: a multicentre point prevalence survey across seven hospitals in Ghana. JAC-Antimicrob Resist 2021; 3: dlab087. 10.1093/jacamr/dlab087 34263166 PMC8275021

[14] Eastern Regional Hospital. https://erhk.org/index.html.

[15] Ghana Statistical Services. https://www.statsghana.gov.gh/regionalpopulation.php?population=MTM5ODc0NTI3OS45NTQ1&&Eastern&regid=5, 2023.

[16] World Health Organization . WHO methodology for a global programme on surveillance of antimicrobial consumption. https://apps.who.int/iris/bitstream/handle/10665/336215/9789240012639-eng.pdf, 2020

[17] WHO . Guidelines for ATC Classification and DDD Assignment. WHO Collaborating Centre for Drug Statistics Methodology, 2013.

[18] Ministry of Health Ghana National Drugs Programme . Standard Treatment Guidelines. *Seventh Edn*. Accra, Ghana, 2017.

[19] Hsia Y , Lee BR, Versporten A et al Use of the WHO access, watch, and reserve classification to define patterns of hospital antibiotic use (AWaRe): an analysis of paediatric survey data from 56 countries. Lancet Glob Health 2019; 7: e861–71. 10.1016/S2214-109X(19)30071-3 31200888

[20] WHO . 2021 AWaRe classification. https://www.who.int/publications-detail-redirect/2021-aware-classification, 2021.

[21] Medicines List. http://www.nhis.gov.gh/medlist.aspx, 2023.

[22] Klein EY , Milkowska-Shibata M, Tseng KK et al Assessment of WHO antibiotic consumption and access targets in 76 countries, 2000–15: an analysis of pharmaceutical sales data. Lancet Infect Dis 2021; 21: 107–15. 10.1016/S1473-3099(20)30332-7 32717205

[23] Adrizain R , Setiabudi D, Chairulfatah A. The inappropriate use of antibiotics in hospitalized dengue virus-infected children with presumed concurrent bacterial infection in teaching and private hospitals in Bandung, Indonesia. PLoS Negl Trop Dis 2019; 13: e0007438. 10.1371/journal.pntd.0007438 31226110 PMC6608981

[24] D’Arcy N , Ashiru-Oredope D, Olaoye O et al Antibiotic prescribing patterns in Ghana, Uganda, Zambia and Tanzania hospitals: results from the global point prevalence survey (G-PPS) on antimicrobial use and stewardship interventions implemented. Antibiotics 2021; 10: 1122. 10.3390/antibiotics10091122 34572704 PMC8469030

[25] Amponsah OKO , Buabeng KO, Owusu-Ofori A et al Point prevalence survey of antibiotic consumption across three hospitals in Ghana. JAC-Antimicrob Resist 2021; 3: dlab008. 10.1093/jacamr/dlab008 34223086 PMC8210176

