# Supplementary material for: Antibiotic consumption trends in Ghana: analysis of six-years pharmacy issue data from a secondary healthcare facility
Source: JAC Antimicrob Resist. 2023 Mar 21;5(2):dlad025. doi: 10.1093/jacamr/dlad025 (PMC10027653; doi:10.1093/jacamr/dlad025)
Supplement: dlad025_Supplementary_Data [file dlad025_supplementary_data.zip › Table S1.docx]

| Table S1: Ranked Watch and Access antibiotics consumed at the Eastern Regional Hospital in Defined Daily Doses per 100 patients | | | | |
| --- | --- | --- | --- | --- |
| Rank | AwaRe antibiotic group | DDD/100 patients | 95% Confidence interval | |
|  |  |  | LL | UL |
| Watch antibiotics | |  |  |  |
| 1 | Cefuroxime | 287.3 | 259.1 | 315.7 |
| 2 | Azithromycin | 88.4 | 75.9 | 100.8 |
| 3 | Ciprofloxacin | 82.9 | 72.1 | 93.7 |
| 4 | Ceftriaxone | 42.0 | 38.9 | 45.0 |
| 5 | Clarithromycin | 39.9 | 28.8 | 51.0 |
| 6 | Erythromycin | 14.1 | 11.1 | 17.0 |
| 7 | Levofloxacin | 5.9 | 4.8 | 7.1 |
| 8 | Cefixime | 3.4 | 0.4 | 6.5 |
| 9 | Meropenem | 0.5 | 0.4 | 0.5 |
| 10 | Cefpodoxime | 0.2 | 0.2 | 0.2 |
| 11 | Cefotaxime | 0.1 | 0.07 | 0.1 |
| 12 | Vancomycin | 0.009 | 0.005 | 0.01 |
| 13 | Ceftazidime | 0.004 | 0.003 | 0.005 |
|  |  |  |  |  |
| Access antibiotics | |  |  |  |
| 1 | Amoxicillin-clavulanate | 372.6 | 354.1 | 391.0 |
| 2 | Sulfamethoxazole trimethoprim | 145.8 | 119.3 | 172.3 |
| 3 | Metronidazole | 127.9 | 119.2 | 136.6 |
| 4 | Doxycycline | 95.9 | 85.2 | 106.6 |
| 5 | Clindamycin | 91.9 | 82.4 | 101.5 |
| 6 | Amoxicillin | 74.86514 | 67.1 | 82.6 |
| 7 | Flucloxacillin | 28.0 | 24.5 | 31.6 |
| 8 | Benzyl penicillin | 18.4 | 16.9 | 19.9 |
| 9 | Gentamicin | 12.7 | 11.7 | 13.7 |
| 10 | Phenoxymethyl Penicillin | 5.9 | 5.0 | 6.7 |
| 11 | Tetracycline | 0.5 | 0.2 | 0.8 |
| 12 | Amikacin | 0.4 | 0.3 | 0.5 |
| 13 | Ampicillin | 0.3 | 0.2 | 0.3 |
| *CI, confidence interval; LL, lower limits; UL, upper limits; DDD, daily defined doses | | | | |
