# Supplementary material for: Antibiotic consumption trends in Ghana: analysis of six-years pharmacy issue data from a secondary healthcare facility
Source: JAC Antimicrob Resist. 2023 Mar 21;5(2):dlad025. doi: 10.1093/jacamr/dlad025 (PMC10027653; doi:10.1093/jacamr/dlad025)
Supplement: dlad025_Supplementary_Data [file dlad025_supplementary_data.zip › Table S2.docx]

| Table S2: Yearly Antimicrobial consumption in Defined Daily Dose per 100 patients at Anatomic Therapeutic Classification Level 4 | | | | | | | |
| --- | --- | --- | --- | --- | --- | --- | --- |
| ATC Level 4 | 2016 | 2017 | 2018 | 2019 | 2020 | 2021 | six-year total |
| Combination of Penicillins including Beta lactamase | 52.4 | 69.9 | 68.7 | 60.5 | 56.9 | 64.3 | 372.6 |
| Second generation cephalosporins | 37.9 | 52.0 | 62.0 | 41.5 | 47.2 | 46.8 | 287.4 |
| Combination of sulfonamides and trimethoprim including derivatives | 47.3 | 58.3 | 40.1 | 0.1 | 0.002 | 0 | 145.8 |
| Macrolide | 15.5 | 22.6 | 21.7 | 25.8 | 17.9 | 38.8 | 142.3 |
| Imidazole derivatives | 24.1 | 20.7 | 20.3 | 19.8 | 25.3 | 17.7 | 127.9 |
| Tetracyclines | 14.3 | 13.3 | 17.7 | 17.2 | 19.3 | 14.6 | 96.4 |
| Lincosamides | 14.8 | 22.5 | 17.0 | 11.0 | 13.0 | 13.7 | 91.9 |
| Fluoroquinolones | 17.5 | 20.3 | 15.0 | 14.8 | 7.8 | 13.5 | 88.9 |
| Penicillins with extended spectrum | 12.1 | 13.9 | 10.1 | 12.5 | 11.9 | 14.7 | 75.1 |
| Third generation cephalosporins | 4.3 | 3.9 | 7.4 | 6.5 | 11.7 | 12.0 | 45.8 |
| Beta lactamase resistant penicillins | 5.2 | 6.1 | 3.4 | 4.5 | 5.3 | 3.6 | 28.0 |
| Beta lactamase sensitive penicillins | 6.4 | 3.0 | 3.8 | 3.6 | 3.1 | 4.4 | 24.3 |
| Aminoglycoside | 3.4 | 2.3 | 1.9 | 1.7 | 1.7 | 2.1 | 13.1 |
| Carbapenems | 0.07 | 0.08 | 0.1 | 0.05 | 0.09 | 0.04 | 0.5 |
| Glycopeptides | 0 | 0 | 0 | 0 | 0.008 | 0.001 | 0.009 |
| Derivatives of Nitroimidazoles | 0 | 0 | 0 | 0 | 0 | 0 | 0 |
| Total | 255.4 | 308.7 | 289.1 | 219.5 | 221.2 | 246.1 | 1540.0 |
| *ATC, anatomical therapeutic classification index | | | | | | | |
