# Supplementary material for: Antibiotic consumption trends in Ghana: analysis of six-years pharmacy issue data from a secondary healthcare facility
Source: JAC Antimicrob Resist. 2023 Mar 21;5(2):dlad025. doi: 10.1093/jacamr/dlad025 (PMC10027653; doi:10.1093/jacamr/dlad025)
Supplement: dlad025_Supplementary_Data [file dlad025_supplementary_data.zip › Table S3.docx]

| Table S3: Yearly Antimicrobial consumption in Defined Daily Dose per 100 patients at Anatomic Therapeutic Classification Level 5 | | | | | | | |
| --- | --- | --- | --- | --- | --- | --- | --- |
| ATC Level 5 | 2016 | 2017 | 2018 | 2019 | 2020 | 2021 | six-year total |
| Amoxicillin-clavulanate | 52.4 | 69.9 | 68.7 | 60.5 | 56.9 | 64.3 | 372.6 |
| Cefuroxime | 37.9 | 52.0 | 62.0 | 41.5 | 47.2 | 46.8 | 287.4 |
| Sulfamethoxazole trimethoprim | 47.3 | 58.2 | 40.1 | 0.1 | 0.001 | 0 | 145.8 |
| Metronidazole | 24.1 | 20.7 | 20.3 | 19.8 | 25.3 | 17.7 | 127.9 |
| Doxycycline | 14.0 | 13.2 | 17.6 | 17.2 | 19.3 | 14.6 | 95.9 |
| Clindamycin | 14.8 | 22.5 | 17.0 | 11.0 | 13.0 | 13.7 | 91.9 |
| Azithromycin | 5.7 | 13.1 | 14.5 | 8.9 | 13.6 | 32.5 | 88.4 |
| Ciprofloxacin | 16.3 | 18.1 | 13.8 | 14.1 | 7.5 | 13.3 | 82.9 |
| Amoxicillin | 12.1 | 13.9 | 10.1 | 12.5 | 11.8 | 14.6 | 74.9 |
| Ceftriaxone | 4.3 | 3.3 | 4.5 | 6.4 | 11.6 | 11.9 | 42.0 |
| Clarithromycin | 2.1 | 5.7 | 4.7 | 16.9 | 4.2 | 6.3 | 39.9 |
| Flucloxacillin | 5.2 | 6.1 | 3.4 | 4.5 | 5.3 | 3.6 | 28.0 |
| Benzyl penicillin | 6.4 | 2.5 | 1.9 | 2.0 | 2.4 | 3.2 | 18.4 |
| Erythromycin | 7.7 | 3.8 | 2.5 | 0.1 | 0.0 | 0.0 | 14.1 |
| Gentamicin | 3.4 | 2.3 | 1.9 | 1.6 | 1.7 | 1.9 | 12.7 |
| Levofloxacin | 1.2 | 2.2 | 1.2 | 0.7 | 0.4 | 0.2 | 5.9 |
| Phenoxymethyl Penicillin | 0.0 | 0.5 | 1.9 | 1.7 | 0.7 | 1.2 | 5.9 |
| Cefixime | 0.0 | 0.6 | 2.8 | 0.0 | 0.0 | 0.0 | 3.4 |
| Tetracycline | 0.3 | 0.1 | 0.1 | 0.1 | 0.0 | 0.0 | 0.5 |
| Meropenem | 0.1 | 0.1 | 0.1 | 0.1 | 0.1 | 0.0 | 0.5 |
| Amikacin | 0.02 | 0.02 | 0.01 | 0.04 | 0.09 | 0.2 | 0.4 |
| Ampicillin | 0 | 0 | 0.07 | 0.06 | 0.05 | 0.07 | 0.3 |
| Cefpodoxime | 0 | 0 | 0.03 | 0.07 | 0.05 | 0.07 | 0.2 |
| Cefotaxime | 0.06 | 0.02 | 0 | 0.03 | 0 | 0 | 0.1 |
| Ceftriaxone beta lactamase combination | 0 | 0 | 0 | 0.004 | 0 | 0.02 | 0.03 |
| Vancomycin | 0 | 0 | 0 | 0 | 0.008 | 0.001 | 0.009 |
| Ceftazidime | 0 | 0.0009 | 0.003 | 0.0007 | 0 | 0 | 0.004 |
| *ATC, anatomical therapeutic classification index | | | | | | | |
